# Profiles of responses of immunological factors to different subtypes of Kawasaki disease

**DOI:** 10.1186/s12891-015-0744-6

**Published:** 2015-10-23

**Authors:** Yan Ding, Gang Li, Li-Juan Xiong, Wei Yin, Jie Liu, Fan Liu, Rui-Geng Wang, Kun Xia, Shu-Ling Zhang, Lei Zhao

**Affiliations:** Department of Rheumatology and Immunology, Medical and Health Center for Women and Children, Wuhan Children’s Hospital, Tongji Medical College, Huazhong University of Science and Technology, Wuhan, 430016 P.R. China; Department of Infectious Diseases, Renmin Hospital, Hubei University of Medicine, Shiyan, 442000 P.R. China; Department of Infectious Diseases, Union Hospital, Tongji Medical College, Huazhong University of Science and Technology, Wuhan, 430022 P.R. China; Department of Critical-Care Medicine, Renmin Hospital, Hubei University of Medicine, Shiyan, 442000 P.R. China

**Keywords:** Kawasaki disease, Mucocutaneous lymph node syndrome, T lymphocyte subsets, Natural killer cells, B lymphocytes, Immunoglobulin, Coronary artery lesion

## Abstract

**Background:**

The responses of immunological factors to different subtypes of Kawasaki disease (KD) remain poorly understood.

**Methods:**

We recruited 388 patients with KD, 160 patients with infectious febrile disease and 85 normal children who served as control subjects. Both the levels and percentages of T lymphocyte subsets, natural killer cells (NK cells) and B cells were analyzed via flow cytometry. The levels of serum IgG, IgM, IgA and C3, C4 were assessed via velocity scatter turbidimetry.

**Results:**

The most significant differences noted between the patients with infectious febrile disease and the normal children were the elevated levels of B cells, C3 and the ratio of CD4/CD8, and the decreased levels of CD8+ T cells and NK cells, as well as the moderate increase in the absolute value of the CD3+ cells. The decreased T cell levels and the elevated B cell levels were helpful in distinguishing typical KD from atypical KD; the elevated T cell levels, the elevated NK cell and B cell levels and the decreased B cell levels were helpful in predicting the effectiveness of IVIG; low C3 and C4 levels were linked with prodromal infections.

**Conclusions:**

Lymphocytes subsets and complement markers may be useful in differentiating among the different subtypes of KD and in helping clinicians understand the pathophysiology of KD.

## Background

Kawasaki disease (KD), also known as mucocutaneous lymph node syndrome, is an autoimmune vasculitis syndrome that occurs in children [1]. Both the prevalence and the incidence of KD are much higher among East Asian children compared with children living in the rest of the world [2]. The primary pathological changes associated with KD include systemic non-specific vasculitis involving both small and medium-sized blood vessels, particularly the coronary arteries, and the possible formation of coronary artery aneurysms, which cause coronary narrowing and thrombotic infarction, resulting in both myocardial infarction and sudden death [3]. KD may cause cardiac complications among children and may increase an individual’s risk of ischemic heart disease during adulthood [4].

Although the pathogenesis of KD has not been fully elucidated, infectious agents are postulated to be responsible for the disease’s onset [5], as both the activation and the dysfunction of the immune system are often observed during the acute phase of the disease [6]. Based on the results of recent research studies, it is possible that the immunopathogenesis of KD is related to acute rheumatic fever. The hypothetical pathogenesis underlying the development of KD is as follows [7]: following an infection involving an unknown KD pathogen, pathogenic substances produced as a result of the infection spread and bind to the endothelial cells of both small and medium-sized blood vessels. To control the actions of these pathogenic substances, the cells of the immune system are activated. Initially, non-specific T cells and non-specific antibodies are involved in this reaction; these hyperactivated immune cells produce various types of cytokines, which results in a cytokine imbalance associated with further endothelial cell injury [7]. Following the resolution of the inflammation involving the blood vessels, specific T cells and antibodies are released against the pathogenic proteins; tissue injury subsequently ceases, and a repair process involving the immune cells begins [7].

Therefore, this hypothesis implies that the lymphocyte levels in patients with KD may be different from those observed in both normal control subjects and febrile patients and that the levels of these lymphocytes may vary among the subtypes of KD. However, the mechanisms underlying the changes involving the T cells, the B cells, the natural killer cells and the immunoglobulins in patients with KD remain unclear; therefore, no accurate diagnostic tests or laboratory tests that may help clinicians differentiate among the subtypes of KD have been developed [6, 8–10]. It is necessary to determine the mechanisms underlying the changes involving IgG, IgM and IgA, C3 and C4 and T cells, B cells and NK cells in the different types of KD to determine the possible role that a particular diagnostic test may have in the setting of KD. In this study, we performed a comparison of the immunological response profiles of children with different subtypes of KD.

## Methods

### Subjects

A total of 388 hospitalized patients with KD (262 boys and 126 girls) between 2 months and 14 years of age were recruited from Wuhan Children’s Hospital, Wuhan, China, from August 2010 to April 2013. Each of the patients had been previously healthy, as their symptoms had first manifested within 10 days of their being hospitalized. Children receiving either hormone therapy or immunosuppressive therapy, children who had suffered from an infectious disease that was diagnosed by a physician and confirmed via laboratory testing within the previous 2 weeks, and children who suffered from another autoimmune disease were not eligible for the study. All study procedures conformed to the ethical standards established by the Medical Ethics Committee of Wuhan Children’s Hospital and were approved by the Commission. All study protocols conformed to internationally accepted principles and the guidelines of the Ethics Committee of Wuhan Children’s Hospital. Informed consent was authorized by the Medical Ethics Committee of Wuhan Children’s Hospital. Consent was provided by the patient’s next of kin, caretaker, or guardian. Each participant or individual acting on their behalf provided written informed consent to participate in this study.

### Controls

The febrile control group included children enrolled from August 2012 to April 2013. A total of 160 patients, including 104 males and 56 females between 2 months and 11 years of age, with an average age of 2.43 ± 2.20 years, were included. The febrile controls included children with febrile diseases caused by respiratory tract infections, including pneumonia, bronchopneumonia, and acute tonsillitis. The normal control group was recruited from January to December, 2013. A total of 85 patients, including 54 males and 31 females between 4 months and 13.17 years of age, with an average age of 2.49 ± 1.89 years, were recruited. The normal controls included patients who presented for routine health examinations, circumcisions or indirect inguinal hernias. No significant difference was noted among the three groups regarding the children’s ages. The age ratios of the male and female participants in each of the control groups were not significantly different.

### The diagnostic criteria for KD

The diagnosis of KD was made using the criteria defined by the American Heart Association [11, 12]. A child was diagnosed with typical KD if he or she had fever for 5 days or more, with at least 4 of the following 5 signs or symptoms: (1) acute changes in the extremities, including erythema and edema of the hands and feet and membranous desquamation of the fingertips; (2) polymorphous exanthema; (3) bilateral, painless bulbar conjunctivitis without exudate; (4) changes in the lips and oral cavity, including erythema and cracking of lips, a strawberry tongue, and diffuse injection of the oral and pharyngeal mucosa; and (5) cervical lymphadenopathy (≥1.5 cm in diameter), which is usually unilateral [11, 12].

### The diagnosis of incomplete KD

The diagnosis of incomplete KD was also made using the criteria of the American Heart Association [11, 12]. A child was diagnosed with incomplete KD (or atypical KD) if he or she had fever for more than 5 days but had fewer than 4 of the 5 clinical manifestations mentioned above.

### The diagnostic criteria of the coronary artery lesions (CAL)

The CAL diagnostic criteria were also defined by the American Heart Association [11, 12], as follows: (1) an ultrasonic cardiograph (UCG) depicted enhancement of the coronary endomembrane; (2) the normal coronary artery diameter and aortic annulus (AoA) diameter ratio for the left coronary artery (LCA)/AoA = 0.150 ± 0.020 (0.09 ~ 0.21) or the right coronary artery (RCA)/AoA = 0.130 ± 0.020 (0.09 ~ 0.20) occupied the aforementioned ranges. This ratio was independent of age, gender, height, weight and body surface area.

### The criteria for a prodromal infection

The general tests for infectious diseases were performed upon each patient’s visit to a physician, including the IgM test for Chlamydia pneumoniae, Legionella pneumophila serotype I, Rickettsia burneti, Influenza type A and B, Parainfluenza, Adenovirus, Respiratory Syncytial Virus, IgM and IgG, Early Antigen and Core Antigen for Epstein-Barr Virus (EBV), IgM and IgG for Cytomegalovirus (CMV) and Mycoplasma pneumonia, the antistreptolysin “o” test (ASO), and a blood culture for both oxybiontic and anaerobic bacteria. If any of the above tests was positive, a prodromal infection was considered.

### The treatment of patients with KD

The IVIG treatment window occurred within 5 to 10 days following the onset of fever. The lyophilized human serum low pH intravenous gamma globulin was produced by the Chengdu Institute of Biological Products at the Ministry of Health. A single intravenous infusion of IVIG was given at a dose of 2 g/kg and combined with aspirin therapy at a dose of 30 ~ 50 mg/(kg • d); these treatments were divided into 3 to 4 doses for continuous treatment until each patient’s symptoms disappeared, as indicated by a normal level of C-reactive protein, a normal platelet count, a normal white blood cell count, and a normal erythrocyte sedimentation rate, after two months had passed. The course of treatment for concurrent CAL included the prolonged administration of aspirin until each patient’s coronary artery diameters returned to their normal values. The patients suffering from concurrent CAL or exhibiting PLT ≥ 500 × 10^9^/L were simultaneously treated with oral dipyridamole, 30 ~ 50 mg / (kg.d).

### The criteria of both sensitive and non-reactive KD for intravenous immunoglobulin (IVIG) therapy

After IVIG treatment was administered, the diagnosis of non-reactive KD was confirmed if the patient’s fever (i.e., body temperature >38 °C) did not disappear within 48 h of the most recent infusion [13]. If the fever resolved, the patient’s KD was diagnosed as IVIG sensitive KD.

### Specimen collection

Four milliliters of venous blood were split into a coagulant tube and an EDTA-tube. Both the immunoglobulins and the lymphocyte subsets were assayed within 8 h. All specimens were collected before beginning IVIG treatment.

### Instruments and reagents

An FACS Calibur flow cytometry instrument equipped with an argon ion laser with an excitation wavelength of 488 nm was manufactured by BD Biosciences. The immunoglobulin detector, a BN-II special protein analyzer, was produced by Siemens. Philips IE33 color Doppler echocardiography was used for the UCG examination, and the probe was an S8-3 child-specific cardiac probe. The CD4-APC, CD8-PE, CD3-FITC, CD19-APC, CD16CD56-PE fluorescent antibody, hemolysin (10×) and absolute count tubes (Trucount tube) were each purchased from BD Biosciences.

### Detection via flow cytometry

Approximately 50 μl of whole blood were aspirated into a Trucount tube, and 20 μl of fluorescent antibody was subsequently added. After 20 min, 450 μl of hemolysin (1×) were added in the dark at room temperature and allowed to remain at room temperature for 15 min before testing. The automatic software Multi TEST was utilized to analyze the lymphocyte subsets.

### Statistical analysis

The differences among three groups were compared via ANVOA, and the differences between two groups were compared using an independent samples *t*-test. SPSS 12.0 statistical software was utilized to complete the statistical analysis. *P* <0.05 was considered statistically significant.

## Results

### Study participants

All 388 patients were recruited from Wuhan Children’s Hospital. As shown in Table 1 and 2, the mean age of the participants was 2.30 years (range: 0.17–14) old, and 32.47 % of the participants were girls. The majority of the patients (*n* = 213, 54.0 %) suffered a prodromal infection. Three hundred and seventy-three patients (96.13 %) were diagnosed with typical KD. One hundred patients (25.78 %) were diagnosed with CAL, and 364 patients (93.81 %) were responsive to IVIG treatment. The incidence rate of KD was higher among male patients than female patients, but the age of onset was not different between the male and female patients.

**Table 1:** Study participants

KD cases	Mean age	Girls %	Prodromal infection	Typical KD	CAL	Respond to IVIG
388	2.30 years	32.47 %	54.0 %	96.13 %	25.78 %	93.81 %

**Table 2:** Demographic details

	KD group		Febrile control		Normal control	
subsets	*N*	Age	*N*	Age	*N*	Age
all cases	388	2.30 ± 1.99	160	2.43 ± 2.20	85	2.49 ± 1.89
male	262	2.25 ± 2.02	104	2.24 ± 2.09	54	2.23 ± 1.35
female	126	2.40 ± 1.93	56	2.79 ± 2.37	31	2.96 ± 2.53
prodromal infection	213	2.18 ± 2.02				
no prodromal infection	175	2.44 ± 1.95				
typical KD	373	2.29 ± 1.98				
incomplete KD	15	2.56 ± 2.23				
CAL	100	2.46 ± 2.17				
Non-CAL	288	2.24 ± 1.93				
IVIG sensitive	364	2.30 ± 1.94				
IVIG nonresponsive	24	2.33 ± 2.71				

### A comparison of the lymphocyte subsets among the KD, febrile and normal control groups

As shown in Table 3, the levels of CD3 cells noted the in KD group were markedly lower than those noted in the other two groups (*p* <0.01), whereas the absolute values in both the KD and the Febrile group were higher than that of that Normal group (*p* <0.01); the Febrile group exhibited the largest elevation (*p* <0.01). Regarding the CD4 cells, both the level and the absolute value were elevated in the KD group compared with the Normal group (*p* <0.01); however, these parameters were not significantly different from those of the Febrile group. Regarding the CD8 cells, the level noted in the KD group was significantly lower than those of the other two groups (*p* <0.01); however, the absolute value was decreased only in the Febrile group (*p* <0.01). The levels of the CD16 and CD56 cells (NK cells) in both the KD and the Febrile groups were significantly lower than those of the Normal group; however, the levels noted in KD group were the lowest of the three groups (*p* <0.01), whereas the absolute values in both the KD and Normal group were notably decreased compared with those of the Febrile group (*p* <0.01). Regarding the B cells, both the level and the absolute value of the CD19 cells in both the KD and the Febrile groups were markedly elevated compared with that of the Normal group (*p* <0.01); the KD group demonstrated the most significant elevations of the two parameters (*p* <0.01 and *p* <0.05). The results regarding the ratio of CD4/CD8 were the same as those noted regarding the B cells (*p* <0.01).

**Table 3:** Comparison of absolute value and percentage of lymphocyte subsets among KD, febrile and normal group

Group	*n*	CD3	CD4	CD8	CD16 CD56	CD19	CD4/CD8
KD group (count/μl)	388	2177.68 ± 1361.88**††	1360.66 ± 914.86**	694.96 ± 492.43††	312.14 ± 291.27††	1218.80 ± 803.16**†	
Febrile group (count/μl)	160	2680.22 ± 2105.36**	1496.28 ± 1179.19**	1068.54 ± 1220.52**	428.56 ± 430.11	1046.73 ± 863.80**	
Normal group (count/μl)	85	1765.88 ± 656.00	890.09 ± 363.65	724.53 ± 299.90	369.38 ± 232.85	539.92 ± 257.89	
KD group (%)	388	56.23 ± 10.50**††	35.09 ± 9.28**	18.12 ± 6.50**††	8.23 ± 5.47**††	32.88 ± 11.27**††	2.27 ± 1.13**††
Febrile group (%)	160	62.08 ± 11.30	34.70 ± 9.08**	24.07 ± 9.27**	10.18 ± 5.66**	25.27 ± 10.51**	1.63 ± 0.71**
Normal group (%)	85	64.09 ± 6.89	32.01 ± 4.66	26.46 ± 4.96	13.45 ± 5.96	19.75 ± 5.00	1.25 ± 0.30

Data were shown as mean ± SD. * *p* <0.05 compared to the normal group, ** *p* <0.01 compared to the normal group; † *p* <0.05 compared to the febrile group, †† *p* <0.01 compared to the febrile group

### A comparison of the immunoglobulins among the KD, Febrile and normal control groups

As shown in Table 4, the level of IgG in the KD group was significantly lower than that of the Febrile group (*p* <0.01) but was not different than that of the Normal group. The levels of IgA in both the KD group and the Febrile group were higher than that of the Normal group (*p* <0.05); however, the KD group’s level was more significant (*p* <0.01), whereas the values of IgM among the three groups were not significantly different. The levels of C3 in both the KD and the Febrile groups were much higher than that of the Normal group (*p* <0.01); however, the KD group exhibited greater elevation compared with the Febrile group (*p* <0.01). Regarding C4, the KD group was not significantly different compared with the Normal group, but the Febrile group exhibited an elevated level compared with the other two groups, a difference that was statistically significant (*p* <0.01 and *p* <0.05).

**Table 4:** Comparison of immunoglobin and complements among KD, febrile and normal group

Group	*n*	IgG	IgA	IgM	C_3_	C_4_
KD group (g/L)	388	7.13 ± 3.00††	0.77 ± 0.49**	1.29 ± 0.64	1.28 ± 0.24**††	0.27 ± 0.09††
Febrile group (g/L)	160	8.02 ± 2.93**	0.75 ± 0.58*	1.28 ± 0.53	1.14 ± 0.28**	0.30 ± 0.13*
Normal group (g/L)	85	7.21 ± 1.48	0.63 ± 0.34	1.15 ± 0.44	1.01 ± 0.21	0.28 ± 0.09

Data were shown as mean ± SD. * *p* <0.05 compared to the normal group, ** *p* <0.01 compared to the normal group; † *p* <0.05 compared to the febrile group, †† *p* <0.01 compared to the febrile group

### A comparison of the lymphocyte subsets and the immunoglobulins between the typical KD group and the incomplete KD group

As shown in Table 5, compared with the incomplete group, the absolute values of the CD3, CD4 and CD8 cells were significantly lower among the patients with typical KD (*p* <0.05), whereas the percentage of the CD3 cells was lower, and the percentage of the CD19 cells was markedly higher among these patients (*p* <0.01). As shown in Table 6, there were no significant differences in the levels of immunoglobulins and complement between the typical KD group and the incomplete KD group.

**Table 5:** Comparison of absolute value and percentage of lymphocyte subsets between typical and incomplete KD

Group	*n*	CD3	CD4	CD8	CD16 CD56	CD19	CD4/CD8
Typical KD (count/μl)	373	2144.26 ± 1355.17*	1341.05 ± 915.06*	683.15 ± 487.67*	306.75 ± 291.09	1220.17 ± 807.47	2.28 ± 1.15
Incomplete KD (count/μl)	15	3008.73 ± 1304.49	1848.13 ± 788.68	988.47 ± 536.45	446.07 ± 271.47	1184.80 ± 710.88	2.03 ± 0.71
Typical KD (%)	373	55.92 ± 10.49**	34.92 ± 9.36	17.99 ± 6.48	8.19 ± 5.53	33.27 ± 11.26**	
Incomplete KD (%)	15	64.00 ± 7.41	39.33 ± 5.61	21.20 ± 6.54	9.27 ± 3.73	23.40 ± 6.41	

Data were shown as mean ± SD. * *p* <0.05 compared to incomplete KD group, ** *p* <0.01 compared to incomplete KD group

**Table 6:** Comparison of immunoglobin and complements between typical and incomplete KD group

Group	*n*	IgG	IgA	IgM	C_3_	C_4_
Typical KD (g/L)	373	7.12 ± 3.05	0.78 ± 0.50	1.29 ± 0.64	1.28 ± 0.25	0.27 ± 0.09
Incomplete KD (g/L)	15	7.39 ± 1.62	0.70 ± 0.35	1.29 ± 0.48	1.25 ± 0.23	0.29 ± 0.08

Data were shown as mean ± SD. * *p* < 0.05 compared to incomplete KD group, ** *p* < 0.01 compared to incomplete KD group

### A comparison of the lymphocyte subsets and the immunoglobulins between the IVIG nonresponsive group and the IVIG sensitive group

As shown in Table 7, compared with the IVIG nonresponsive group, the absolute values of the CD3, CD4, CD8, CD16CD56 and CD19 cells were significantly higher among the patients in the IVIG sensitive group (*p* <0.01 and *p* <0.05), and the percentages of the CD3, CD4, CD8 and CD19 cells were markedly higher in this group (*p* <0.01 and *p* <0.05). As shown in Table 8, the levels of C3 were significantly higher among the patients of the IVIG sensitive group (*p* <0.05).

**Table 7:** Comparison of absolute value and percentage of lymphocyte subsets between IVIG sensitive and nonresponsive group

Group	*n*	CD3	CD4	CD8	CD16 CD56	CD19	CD4/CD8
IVIG sensitive (count/μl)	364	2235.66 ± 1334.14**	1396.26 ± 906.23**	713.22 ± 480.79**	320.29 ± 287.96*	1240.26 ± 808.21*	2.25 ± 1.12
IVIG nonresponsive (count/μl)	24	1298.38 ± 1501.81	820.75 ± 892.87	417.92 ± 588.64	188.49 ± 319.00	893.38 ± 653.29	2.48 ± 1.29
IVIG sensitive (%)	364	56.80 ± 10.08**	35.39 ± 9.21*	18.34 ± 6.43**	8.37 ± 5.53	32.23 ± 10.75**	
IVIG nonresponsive (%)	24	47.71 ± 13.04	30.50 ± 9.19	14.75 ± 6.73	6.18 ± 4.03	42.83 ± 14.25	

Data were shown as mean ± SD. * *p* <0.05 compared to IVIG nonresponsive group, ** *p* <0.01 compared to IVIG nonresponsive group

**Table 8:** Comparison of immunoglobin andcomplements between IVIG sensitive and nonresponsive group

Group	*n*	IgG	IgA	IgM	C_3_	C_4_
IVIG sensitive (g/L)	364	7.11 ± 2.86	0.77 ± 0.48	1.28 ± 0.54	1.29 ± 0.24*	0.27 ± 0.09
IVIG nonresponsive (g/L)	24	7.50 ± 4.79	0.76 ± 0.61	1.47 ± 1.48	1.14 ± 0.28	0.24 ± 0.08

Data were shown as mean ± SD. * *p* <0.05 compared to IVIG nonresponsive group, ** *p* <0.01 compared to IVIG nonresponsive group

### A comparison of the lymphocyte subsets and the immunoglobulins between the CAL group and the non-CAL group

As shown in Tables 9 and 10, there were no differences in the levels of either the lymphocyte subsets or the immunoglobulins between the CAL group and the non-CAL group.

**Table 9:** Comparison of absolute value and percentage of lymphocyte subsets between CAL and non-CAL group

Group	*n*	CD3	CD4	CD8	CD16 CD56	CD19	CD4/CD8
CAL (count/μl)	100	2067.04 ± 1369.82	1296.81 ± 903.35	630.54 ± 481.71	269.18 ± 243.26	1239.31 ± 896.90	2.40 ± 1.35
Non-CAL (count/μl)	288	2216.10 ± 1359.39	1382.83 ± 919.34	717.32 ± 494.96	327.05 ± 305.16	1211.68 ± 769.50	2.22 ± 1.05
CAL (%)	100	55.42 ± 11.00	34.86 ± 8.99	17.21 ± 6.80	7.45 ± 4.57	34.50 ± 12.95	
Non-CAL (%)	288	56.52 ± 10.32	35.17 ± 9.39	18.43 ± 6.37	8.50 ± 5.73	32.32 ± 10.59	

Data were shown as mean ± SD. * *p* <0.05 compared to non-CAL group, ** *p* <0.01 compared to non-CAL group

**Table 10:** Comparison of immunoglobin and complements between CAL and non-CAL group

Group	*n*	IgG	IgA	IgM	C_3_	C_4_
CAL (g/L)	100	7.40 ± 3.25	0.80 ± 0.48	1.43 ± 0.91	1.27 ± 0.25	0.26 ± 0.08
Non-CAL (g/L)	288	7.04 ± 2.91	0.76 ± 0.49	1.24 ± 0.50	1.29 ± 0.24	0.28 ± 0.09

Data were shown as mean ± SD. * *p* <0.05 compared to non-CAL group, ** *p* <0.01 compared to non-CAL group

### A comparison of the lymphocyte subsets and the immunoglobulins between the infection group and the non-infection group

As shown in Tables 11 and 12, compared with the non-infection group, the levels of C3 and C4 were significantly higher in the infection group (*p* <0.01).

**Table 11:** Comparison of absolute value and percentage of lymphocyte subsets between infection and non-infection group

Group	*n*	CD3	CD4	CD8	CD16 CD56	CD19	CD4/CD8
Infection (count/μl)	213	2220.21 ± 1453.05	1407.65 ± 980.84	706.04 ± 520.13	290.24 ± 273.03	1231.98 ± 831.04	2.29 ± 1.13
Non-infection (count/μl)	175	2125.92 ± 1244.10	1303.46 ± 826.67	681.47 ± 457.58	338.78 ± 310.76	1202.77 ± 769.93	2.24 ± 1.13
Infection (%)	213	56.39 ± 10.60	35.38 ± 9.33	18.05 ± 6.42	7.92 ± 5.72	33.15 ± 11.49	
Non-infection (%)	175	56.05 ± 10.40	34.74 ± 9.23	18.20 ± 6.61	8.60 ± 5.15	32.56 ± 11.01	

Data were shown as mean ± SD. * *p* <0.05 compared to non-infection group, ** *p* <0.01 compared to non-infection group

**Table 12:** Comparison of immunoglobin andcomplements between infection and non-infection group

Group	*n*	IgG	IgA	IgM	C_3_	C_4_
Infection (g/L)	213	7.01 ± 3.20	0.75 ± 0.50	1.27 ± 0.53	1.25 ± 0.23**	0.26 ± 0.08**
Non-infection (g/L)	175	7.28 ± 2.75	0.80 ± 0.48	1.32 ± 0.74	1.32 ± 0.26	0.29 ± 0.09

Data were shown as mean ± SD. * *p* <0.05 compared to non-infection group, ** *p* <0.01 compared to non-infection group

## Discussion

Kawasaki disease was first described in Japan in 1967 and is most common among children in Asian and Pacific Island countries, although it affects children of all ethnicities and races. To date, making an accurate diagnosis remains critical in managing KD; however, the etiology of KD is unknown, and no accurate diagnostic tests or laboratory tests have been developed [14]. Therefore it is necessary to analyze clinical data as a means of developing a diagnostic test for KD. In our study, we analyzed 388 patients with KD, as well as 160 patients with febrile disease and 85 normal control subjects, to analyze the differences in the absolute values and the percentages of the lymphocyte subsets, immunoglobulins and complement types. The data indicated that no differences existed among the three groups regarding the patients’ ages, nor were any differences regarding male and female gender noted among the groups.

The results of this study indicate that the immune statuses of the patients with KD patients were abnormal and that differences in the amounts of specific types of immune cells were noted when compared with either the normal or the febrile control subjects. With the onset of either KD or another febrile disease, both the absolute values and the levels of the CD4+ cells were elevated, which suggested that the helper T cells were activated in response to elevated body temperatures. Regarding the CD8+ cells, the febrile group exhibited a greater degree of elevation; however, the patients with KD exhibited the opposite trend, which indicated that the changes in the levels of the CD8+ cells may represent a distinction between the KD group and the febrile group. Furthermore, the ratio of CD4/CD8 was significantly different among the patients with KD, the infectious febrile patients and the normal children. By contrast, the CD16 and CD56+ cells (NK cells) in KD group exhibited decreased levels, particularly where the percentages of the NK cells were concerned. The CD19+ cells (B cells) were another distinguishing feature of the patients with KD. When the body temperature increased, the percentages and absolutes value of the B cells became elevated in the patients with KD compared with the patients in the infectious febrile group, which demonstrated that elevated B cell levels may be a characteristic used to distinguish these patients from both normal and infectious febrile patients.

We also investigated changes in the levels of the immunoglobulins and the complement types among the patients with KD. The results indicated that the level of IgG was significantly higher in the infectious febrile group compared with the other groups; however, the level of IgM was not characterized by the same pattern, which demonstrated that elevated IgG levels were characteristic of infectious febrile diseases. Regarding the levels of IgA, both the KD group and the febrile group exhibited markedly elevated levels compared with the normal group; however, there was no significant difference between the KD group and the Febrile group, which implied that the levels of IgA may rise both in the setting of KD and in the setting of infectious febrile diseases, and may therefore not be a distinctive marker of either pathology. Regarding the complement types, the level of C4 followed the same trend as did IgG. By contrast, the level of C3 was significantly elevated among the patients with KD patients, more so than among the patients in the infectious febrile group, which demonstrated that the C3 levels may be a parameter used to distinguish KD from other diseases.

The results of these comparisons suggested that the most typical differences between the patients with KD and both the normal children and the infectious febrile patients were the levels of the B cells, C3 and ratio of CD4+ to CD8+ cells. The percentages of the CD8+ T cells and the NK cells were also indicative of differences among the groups, but this relationship did not apply to their absolute values. The levels of the CD3+ T cells were also significantly different among the three groups, but the absolute value of the CD3+ cells in infectious febrile group was much higher than that of the normal group, whereas the percentage was not significantly different from that of the normal group. These results implied that the focus of future KD research may involve B cells, C3, and the CD4/CD8 ratio, as well as the percentages of CD8+ T cells and NK cells; the absolute value of CD3+ cells may also be useful in defining appropriate diagnostic and laboratory tests for KD.

The pathogenesis of KD remains unknown. Previous research has suggested that several single nucleotide polymorphisms may associated with a genetic susceptibility to KD, including functional polymorphisms of the FC gamma RIIa locus and the ITPKC gene [15]. The ITPKC gene is a T-cell activity modulator, which suggests that T cell activity regulation may be an underlying mechanism affecting both an individual’s predisposition to developing KD and the severity of the disease’s course [6]. Our research indicated that the absolute value of the CD3+ cells and the ratio of CD4 to CD8 are distinguishing features of patients with KD. However, the genes encoding B cells and C3 may also be associated with the pathogenesis of KD.

Recent studies have determined that the predomination of IgA plasma cells within mucosal surfaces is a common characteristic of many of the infectious diseases of childhood, as well as KD [8, 16, 17], which suggests that an immune response in a genetically susceptible patient following exposure to a pathogen represents the primary pathogenesis of KD. However, no studies have confirmed this hypothesis [18]. Our research determined that among patients with KD, the levels of the B cells and C3 were both elevated compared with those of normal children and patients with infectious febrile diseases, which demonstrated that infection may be only a trigger for the development of KD and that the primary pathogenesis of the disease may involve the immune system. The elevated B cell levels and elevated C3 levels may indicate that the humoral immune response predominates in patients with KD and that the combination of a lack of cellular immunity and an elevated complement level causes the disease.

We compared the levels of the lymphocyte subsets and the complement types between patients with typical KD and patients with incomplete KD, patients who were IVIG nonresponsive and patients who were IVIG sensitive, CAL and non-CAL subgroups and patients who experienced a prodromal infection and patients who did not experience infection to identify more clinical features with which to differentiate between these subgroups.

Incomplete KD describes patients with an incomplete presentation of the disease, irrespective of the presence of coronary complications, who do not fulfill the classical diagnostic criteria for KD. Comparisons of complete blood counts, albumin levels, hepatic transaminases, and acute phase reactants between the children with typical KD and the children with incomplete KD yielded no significant differences; however, low levels of serum alanine aminotransferase and gamma glutamyltransferase, as well as low frequencies of hyponatremia and pyuria, were reported among the patients with incomplete presentations [19]. However, distinguishing incomplete KD from typical KD remains tied to patients’ clinical presentations as opposed to their laboratory values. Therefore, we compared the lymphocyte subsets, immunoglobulins and complement types between the patients with incomplete KD and the patients with typical KD to find candidates for the laboratory-based differentiation of the subsets of KD. The absolute values of the CD3+, CD4+ and CD8+ T cells were much lower in the typical KD group, which implied that T cell-mediated immune reactions do not predominate in the setting of typical KD. B cell-mediated immune reactions appear to be the primary mechanisms underlying the pathogenesis of KD, as the percentage of CD19+ cells was markedly elevated in the typical KD group. These data demonstrate that the low expression levels of T cells and the high expression levels of B cells may be associated with typical KD. Recent studies have focused on the regulation of T-cell activation as a critical factor in determining an individual’s susceptibility to Kawasaki disease, as well as the severity of an individual’s disease [6]. However, incomplete KD is characterized by a higher T-cell level, which implies that T cell dysregulation plays a role in this disease. It also represents a means with which to differentiate incomplete KD from typical KD.

The treatment for KD is IVIG plus aspirin within 10 days of the onset of symptoms to reduce the risk of coronary artery complications [20]. Therefore, we used IVIG with aspirin to treat KD and attempted to determine if there were any differences between the IVIG sensitive group and the IVIG nonresponsive group. In the IVIG sensitive group, the levels of each of the T cell subgroups, the B cells and the NK cells, but not the ratio of CD4/CD8, were significantly higher than those of the IVIG nonresponsive group, which demonstrates that the immunological responses in the body were activated. Likewise, the percentages of the CD3+, CD4+ and CD8+ cells were significantly increased, and the percentage of the CD19+ cells was significantly decreased in the IVIG sensitive group, which implied that in the IVIG sensitive group, the T-cell-mediated immune responses predominated. Therefore, we can deduce that with increases in the levels of T cells, KD will be more sensitive to IVIG treatment, regardless of the parameters’ absolute values or percentages. There have not been any reports regarding the specific characteristics of KD patients that may make them more likely to be sensitive to IVIG. Our research may help in the identification of markers that are predictive of IVIG sensitivity. Additionally, IVIG resistant KD may be associated with other processes, as opposed to only T-cell-mediated immunological reactions.

KD is a type of systemic vasculitis syndrome that primarily invades the medium-sized muscular arteries, particularly the coronary arteries. Coronary arteritis results in the immediate inflammation of each of the arterial layers following the onset of KD; the inflammation subsequently spreads throughout the artery [5]. We analyzed the lymphocyte subsets and the immunoglobulins between the CAL and the non-CAL group. There were no differences between the groups, which implied that how to predict onset of arteritis may be associated with another type of indication.

Although the underlying mechanism of KD is unknown, epidemiological data suggest that the disease is inked with the increased activation of the immune system in response to infection [14, 21]. However, the exact mechanism is unknown. In this study, we compared the lymphocyte subsets and the immunoglobulins between the patients who suffered a prodromal infection and the patients who did not suffer an infection. The results demonstrated that only the levels of C3 and C4 were lower in the infection group than in the non-infection group. C3 and C4 are the most commonly measured complement components and help the body’s immune system to react to both inflammation and infection. Decreased complement levels are often associated with an increased risk of developing autoimmune diseases or developing recurrent microbial infections [22]. Therefore, the patients with KD who suffered from prodromal infections may also have suffered from autoimmune reactions. Further investigation of this issue is warranted.

For this study, we recruited 388 patients with KD, as well as 160 infectious febrile patients and 85 normal children who served as control subjects, to explore the differences among these groups’ lymphocyte subsets, immunoglobulins and complement types. The results indicated that the differences that distinguished the patients with KD from the infectious febrile patients and the normal children pertained to the B cells, C3 and the ratio of CD4/CD8, as well as the percentages of CD8+ T cells and NK cells and the absolute value of the CD3+ cells. We also compared the profiles of both the lymphocyte subsets and the immunoglobulins between the patients with typical KD and the patients with incomplete KD, and the patients in the IVIG sensitive group and the patients in the IVIG nonresponsive group, the CAL and the non-CAL group, and the patients who suffered a prodromal infection and the patients who did not suffer an infection. These comparisons provided evidence that decreased T cell levels and elevated B cell levels distinguished typical KD from incomplete KD and that elevated T cell levels, NK cell levels and B cell levels, as well as decreased B cell percentages, may be helpful in predicting IVIG effectiveness, whereas low C3 and C4 levels may be associated with prodromal infections. Lymphocyte subsets and complement types may be markers with which to differentiate KD from other infectious febrile diseases and from normal children, and may also be helpful in distinguishing between typical KD and incomplete KD, IVIG sensitivity and IVIG unresponsiveness and patients with prodromal infections and those without prodromal infections. These findings may help to guide the diagnosis of KD and to assist in the differentiation of KD from other diseases, as well as to understand the underlying pathophysiology of the various subtypes of KD.

## Conclusions

Lymphocytes subsets and complements might be some markers for differentiating KD from other infectious febrile diseases and normal children, and also helpful to distingush incomplete KD, IVIG nonresponse and prodromal infection of KD from the status of typical KD, IVIG sensitive and no prodromal infection. These findings shall help to guide diagnosis and differentiation of KD and understand disease pathophysiology of various subtypes of KD.
